# The Silver Medal

**Published:** 1864-10

**Authors:** 


### Correspondence.

### THE SILVER MEDAL.

Eds. Register: The object of this communication is to call the attention of Dentists, manufacturers, and inventers to the proposition of the Mississippi Valley Dental Association to award a silver medal to the person making the most valuable *discovery*, *improvement*, or *invention* pertaining to Dentistry during the present year. I believe that there has not been anything said or written since the publication of the proceedings of the Association on the subject, and unless the exchanges of the *Register* have made mention of the proposition, the probabilities are that very few persons have ever heard that such a prize was to be awarded.

It is said that the pen is mightier than the sword. Then we want the pen used in our behalf. Tell every body that the Missiasippi Valley Association of Dental Surgeons are offering two medals, one to be worth one hundred dollars (gold) for the best essay on Anæsthesia, and the other for the best improvement in anything pertaining to Dentistry.

It seems to me that all medical and scientific journals, as well as other publications, friendly to the advancement of science and art, should give us their aid in our undertaking. We desire that our profession shall rank A No. 1; to accomplish which requires much sacrifice and labor. Now, what we want is to have the co-operation of the person placing this proposition before those interested. Then we feel no hesitancy in saying, that the committee appointed to make the award will have something before them.

If I understand the design, it is not to confine the award to any particular branch of dentistry, but to let anything, it matters not what it is if it is, of utility in the profession, come in for consideration. It matters not whether it is a chain or an "Automatic Plugger," a new method of mounting artificial teeth, or a new anæsthetic agent. All are alike of interest to the committee, and of relative value to the profession. This proposition is not confined to those engaged in the dental profession, but is to any body, whether he be *dentist*, *physician*, or *mechanic.* Can't we have the assistance then of all those interested in these branches of business.

The committee are desirous of making this an important item in the proceedings of the next meeting, which is to be held in February next, to which we invite the attention of all the members of the Association and Profession. If you want to see something new and useful, come to the next meeting. For any particulars in reference to the prize, address Drs. A. S. Talbott or S. Driggs, at Lexington, Ky., or Dr. Shadoan, Box 2079, Cincinnati, Ohio, who will take pleasure in giving any information in their possession.

